# Dentistry Follows the Flag

**Published:** 1900-10

**Authors:** 


### DENTISTRY FOLLOWS THE FLAG.

It is a recognized fact that the school-house follows American civilization. When the pioneer has cleared the forest and prairie, his second thought is to educate his children, and the school-house is built. It was left for recent years to demonstrate that the dental practitioner follows the school-house.

While Congress is discussing, without apparent end, the advisability of having dentists in the army, dentists have not waited for red tape and prejudice, but have quietly advanced with the army, and we find them taking their place in the recently acquired possessions of the United States.

The following programme lies on our desk, and so distinctly represents the character of American dental practitioners that it is deemed worthy a prominent place on our pages. It is as follows:

### MANILA DENTAL SOCIETY.

(Organized February 4, 1900.)

### Officers for 1900-1901.

President .................. Frank R. Harkinson.
Vice-President ............. Juan Arévalo.
Secretary .................. Lloyd R. Hawley.
Treasurer .................. R. L. Hale.

The Society meets on the second Monday of each month (except July and August), at 8 P.M. Legal dental practitioners in the Philippine Islands are eligible to membership. Resident physicians and visiting dentists are always cordially welcome.

### Programme for 1900-1901.

1900.

*March 12.*—Paper by Dr. Louis Ottofy, "Observations on the Practice of Dentistry in the Philippines."

*April 2.*—General Discussion. Incidents of Office Practice.

*May 14.*—General Discussion. Incidents of Office Practice.

*June 11.*—Paper by Dr. W. G. Skidmore, "Dental Laws and Dental Legislation in the Philippines."

*September 10.*—Paper by Dr. Lloyd R. Hawley, "Pyorrhœa Alveolaris."

*October 8.*—Paper by Dr. R. L. Hale, "Gold Filling."

*November 12.*—Paper by Dr. E. B. Merchant, "Crown- and Bridge-Work."

*December 10.*—Paper by Dr. Juan Arévalo, "History of Dentistry in the Philippines."

1901.

*January 14.*—Paper by Dr. Anna M. Sawyer, "Amalgam Filling."

*February 11.*—Annual Address by the President, Dr. Frank R. Harkinson. Annual Meeting, Election of Officers, Reports, etc.

It will be observed that all arrangements for a well-ordered society is in this programme, and it reflects credit upon the representatives of dentistry in this distant region. Some of the names have a familiar sound. The "eternal feminine" is among them.

Thirty-four years ago the writer advocated, in a public address, the admission of woman into dentistry. In the comparatively brief period since, woman has made herself felt in dentistry in all portions of advanced civilization, and now we find her at work in the Philippines, long before the United States Commission has succeeded in convincing the natives that the principles of the American republic would be to their interest to adopt.

The congratulations of the INTERNATIONAL DENTAL JOURNAL are extended to the Manila Dental Society.

